# 

**Published:** 1903-06-15

**Authors:** 


### CONTENTS.

Variations in the Development and Density of Tooth Tissue, - - - *By J. Taft, M.D., D.D.S.* 271

Some Pathological Lesions of the Dental Pulp, and How to Treat Them, - *By Geo. W. Cook, B. S., D.D.S.* 283

Practical Methods in Porcelain Fillings, *By Henry C. Raymond, D.D.S.* 288

Army Dental Surgeon and His Work in the Philippines *By G. D. Boak, D.D.S.* 301

A Case of Cocain Poisoning, - - - *By H. Prinz* 310

Method of Taking Impressions - - - - - 312

Gutta-Percha Cement, - - *By Emil Herbst, D.D.S.* 313

Editorial, - - - - - - - - - 314

Announcements, - - - - - - - - 315

Indents, - - - - - - - - - 319

